# Protocol-aware epidemic forecasting across heterogeneous public health surveillance systems

**DOI:** 10.3389/fpubh.2026.1829302

**Published:** 2026-05-29

**Authors:** Yihan Hu, Jingyuan Han, Mingxin Liu

**Affiliations:** 1 Medical Research Council (MRC) Epidemiology Unit, School of Clinical Medicine, Institute of Metabolic Science, University of Cambridge, Cambridge, United Kingdom; 2 Department of Cancer Prevention and Control, National Cancer Center, Chinese Academy of Medical Sciences and Peking Union Medical College, Beijing, China; 3 Department of Health Communication, Graduate School of Medicine, The University of Tokyo, Tokyo, Japan

**Keywords:** data revisions, epidemic forecasting, health information systems, protocol-aware modeling, public health surveillance, reporting delays and backfill

## Abstract

**Purpose:**

Public-health forecasting is central to epidemic intelligence and operational decision support. In practice, surveillance data are affected by reporting delays, revisions, and backfill, as well as abrupt regime shifts, which often reduce model reliability across regions and systems.

**Methods:**

We developed EpiMap-LLM, a protocol-aware forecasting approach that links epidemic dynamics with surveillance context using a frozen language-model backbone and lightweight trainable components.

**Results:**

Across daily and weekly surveillance settings (JHU CSSE COVID-19 and CDC influenza hospitalization surveillance), EpiMap-LLM consistently improves MAE and RMSE over strong forecasting baselines.

**Conclusion:**

Protocol-aware forecasting improves robustness and practical usefulness for surveillance dashboards, early warning, and public-health decision support in heterogeneous reporting systems.

## 1 Introduction

Public-health agencies increasingly rely on real-time forecasts to support epidemic intelligence and operational decision-making. In practice, forecasts are used in surveillance dashboards and risk-monitoring workflows rather than as standalone numbers. They can trigger surge planning, staffing adjustments, and resource allocation under time pressure and incomplete information. In operational settings, these forecasts directly inform early warning, outbreak preparedness, and near-term resource deployment decisions. To make these forecasts actionable and comparable, the field has built shared infrastructures that aggregate heterogeneous signals, standardize targets, and support systematic evaluation (e.g., COVID-19 and seasonal influenza forecasting) (1–4). Large-scale public-health emergencies (e.g., COVID-19) have further stress-tested these pipelines, exposing both the value of forecasting for coordination and the operational fragility of end-to-end systems at scale (5). Despite sustained methodological progress, producing forecasts that remain dependable under real-world data irregularities and deployment constraints—and that can be trusted as decision-grade inputs within health information systems—continues to be a major challenge (2, 5).

A core challenge is that surveillance time series are shaped as much by reporting protocols as by epidemiological dynamics. Data are often right-truncated and later revised, creating systematic reporting delays and backfill that distort the real-time signal available to a forecasting model (3, 6). From a health information perspective, these streams are inherently versioned: preliminary releases are updated as additional reports arrive and validation processes are completed, which creates a moving target for model supervision and complicates comparability across jurisdictions and systems. In addition, outbreaks are strongly non-stationary: interventions, behavior changes, and pathogen evolution can induce regime shifts that invalidate assumptions learned from earlier phases and exacerbate cross-region distribution shift (5). For deployment, models must therefore separate epidemiological signals from protocol-induced artifacts, remain transferable across regions and temporal resolutions, and behave reliably when data definitions and reporting practices differ across surveillance systems.

Methodologically, prior work spans mechanistic models, statistical nowcasting, and modern deep learning. In this work, our focus is on protocol-aware forecasting under heterogeneous surveillance systems, rather than on building a dedicated nowcasting framework for delay correction or revision reconstruction. Recent work includes long-horizon Transformers, continuous-time neural models, and foundation models with improved transfer performance (7–13). However, two gaps remain under-addressed for public-health use. First, many models treat surveillance streams as purely numerical objects and lack an explicit representation of surveillance semantics (e.g., cadence, spatial hierarchy, and revision/backfill patterns), which often drives brittleness across datasets and limits portability across reporting regimes. Second, scale alone does not guarantee robustness when dominant variations arise from institutional reporting processes rather than underlying disease dynamics; in such settings, models may fit protocol artifacts that do not generalize and can undermine operational trust. These challenges are further amplified when long numeric histories are converted to text, where formatting choices, context limits, and auditability constraints can add instability in deployment.

To address these challenges, we propose EpiMap-LLM, which links numerical epidemic dynamics with explicit surveillance semantics, without serializing long numerical histories into text. EpiMap-LLM includes:

- Temporal State Encoder: a causal-masked time-series encoder that compresses multivariate surveillance trajectories into a compact sequence of latent epidemic state tokens, capturing short- to mid-range dynamics while accommodating heterogeneous reporting pipelines.
- Semantic Prompt Adapter (SPA) : a lightweight, trainable adapter that maps latent epidemic state tokens to continuous prompt embeddings, so a frozen LLM backbone can condition on structured state information instead of brittle text templates.
- Protocol Anchor Bank: a learnable anchor repository that encodes reporting semantics (e.g., temporal cadence, spatial granularity, and backfill/revision patterns). The resulting protocol-aware tokens are injected as context or soft constraints to help distinguish protocol-induced fluctuations from genuine epidemiological shifts, improving transfer across datasets and temporal granularities.

Our main contributions are summarized as follows:

- We formulate epidemic forecasting under heterogeneous surveillance pipelines as a semantic-interface problem, where reliable generalization depends on representing epidemic dynamics together with reporting-process semantics such as cadence, revisions, and backfill.
- We introduce EpiMap-LLM, a parameter-efficient alignment framework that connects numerical time-series representations with frozen LLM embeddings via a Semantic Prompt Adapter (SPA) and a Protocol Anchor Bank, enabling protocol-aware forecasting without full-history text serialization.
- Extensive experiments on representative public-health forecasting benchmarks (e.g., JHU CSSE COVID-19 and CDC influenza hospitalization surveillance) demonstrate consistent improvements over strong forecasting baselines under MAE/MSE, and validate the value of combining semantic prompting with protocol anchoring for robustness under heterogeneous reporting systems and distribution shift.

## 2 Methods

Due to space limitations, we present only a high-level description of the method in the main text. Full technical details (including formulation, module design, optimization objective, implementation settings, and evaluation protocol) are provided in Supplementary material under the same section title, Methods.

### 2.1 Overall architecture

To preserve temporal information and avoid brittle text serialization, EpiMap-LLM is designed as a hybrid architecture rather than either a pure time-series predictor or a text-based LLM forecaster in the usual sense. Its key idea is to combine: (i) a lightweight temporal modeling component for extracting epidemic dynamics from raw surveillance trajectories, (ii) lightweight alignment and protocol-aware modules that translate task-specific numerical information into the LLM space, and (iii) a frozen LLM backbone that serves as the semantic integration layer over these heterogeneous representations.

**Figure 1:**
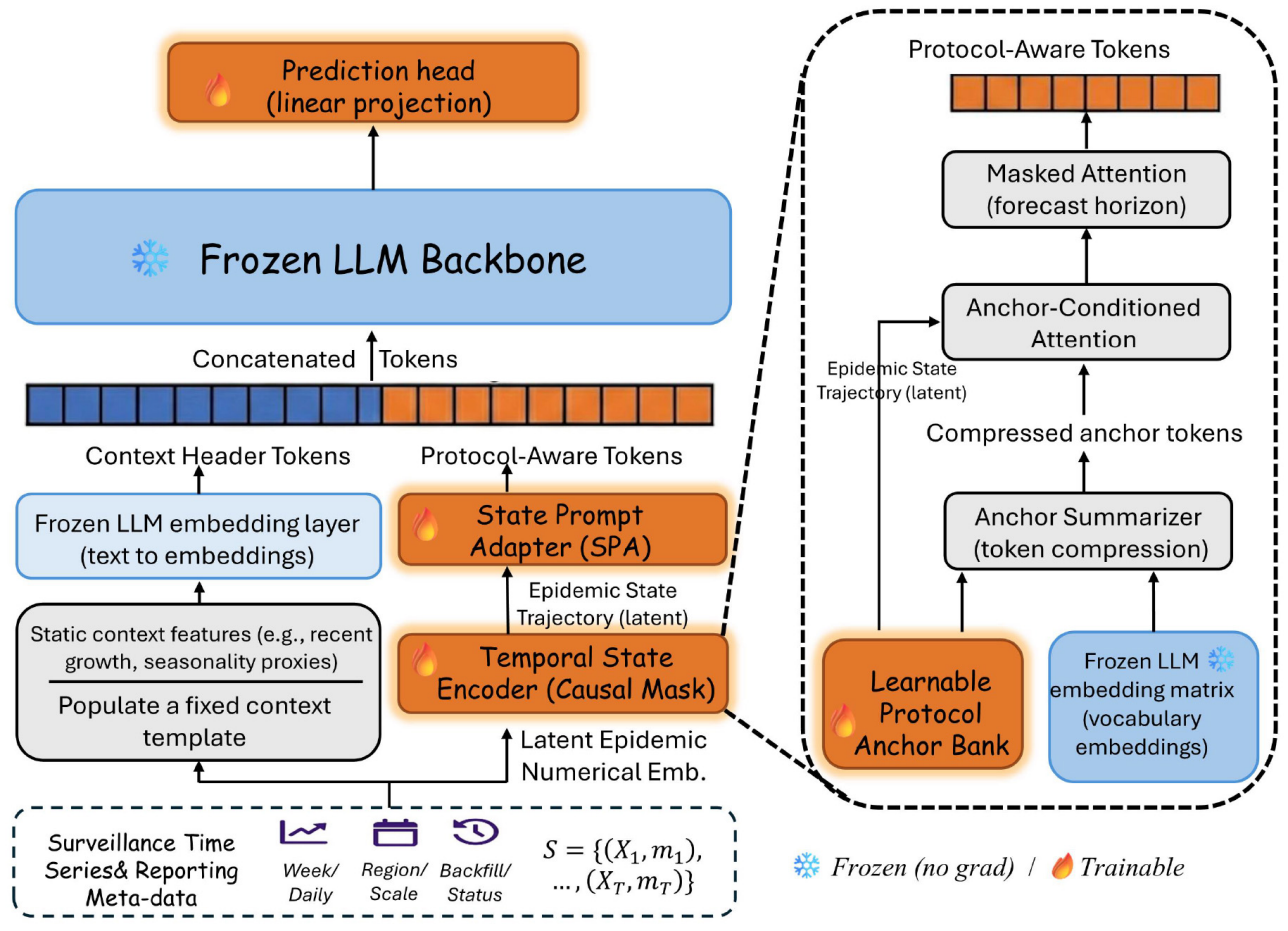
Overview of EpiMap-LLM. A temporal state encoder extracts latent epidemic states from surveillance trajectories; a state prompt adapter and protocol anchor bank inject state and reporting semantics into a frozen LLM for forecasting. Snowflake/flame denote frozen/trainable modules.

To preserve temporal information and avoid brittle text serialization, EpiMap-LLM treats forecasting as a protocol-aware interface problem. The model separates epidemiological dynamics from reporting artifacts rather than treating protocol effects as generic noise. As illustrated in Figure 1, EpiMap-LLM includes three trainable modules that interface with the frozen backbone: (i) a Temporal State Encoder that compresses multivariate trajectories into time-aligned latent epidemic state tokens, thereby performing the primary task-specific numerical modeling of epidemic dynamics; (ii) a State Prompt Adapter (SPA) that maps latent states into continuous prompt embeddings compatible with the frozen embedding space, thereby acting as a semantic alignment module between the temporal encoder and the frozen LLM; and (iii) a Protocol Anchor Bank that produces protocol-aware tokens encoding reporting semantics (e.g., cadence, spatial granularity, and revision/backfill patterns), thereby making the reporting context explicitly available within the same LLM-compatible representation space. In addition, we construct a short, fixed Context Header from static context features (e.g., recent growth and seasonality proxies) and embed it through the frozen input embedding layer, yielding Context Header Tokens. The frozen backbone then integrates these token types through its pretrained embedding and attention space. Accordingly, the role of the LLM in our framework is not conventional text prompting or explicit natural-language reasoning, but high-level semantic integration over continuous prompt embeddings and protocol-aware tokens. The frozen backbone integrates the concatenated tokens via self-attention, and a lightweight Prediction Head produces multi-horizon forecasts.

## 3 Results

### 3.1 Overall forecasting performance

Table 1 summarizes the main results on daily COVID-19 reporting (JHU) and weekly influenza hospitalization surveillance (CDC-IHA). The comparison set is organized around representative forecasting architecture families with different inductive biases, rather than a complete taxonomy of revision-aware or nowcasting-specific public-health methods.

- Consistent gains across reporting regimes. EpiMap-LLM performs best on both datasets, reducing RMSE/MAE by 10.5%–14.8% relative to the strongest baseline, ContiFormer (11). The improvement holds across daily and weekly settings, suggesting that explicitly modeling reporting protocols complements advances in temporal representation learning.
- Protocol-aware conditioning improves robustness beyond irregular-time modeling. Continuous-time baselines (ContiFormer, ODE-RNN (9), and Neural-CDE (10)) benefit from handling irregular dynamics, but within our forecasting setting they still degrade when revisions and backfill introduce protocol-driven fluctuations. By incorporating cadence and revision/backfill semantics through the Protocol Anchor Bank, EpiMap-LLM better separates administrative artifacts from epidemiological changes, which is particularly important for versioned surveillance streams.
- Sequence-only Transformers are sensitive to revision-heavy surveillance. Informer (7) and Autoformer (14) are competitive on the daily benchmark but deteriorate on CDC-IHA, where backfill is pronounced. Conditioning the frozen backbone on protocol-aware tokens mitigates this sensitivity compared with purely sequence-based attention, improving reliability under revision-driven noise.
- General-purpose foundation forecasters show a domain mismatch in surveillance settings. TimesFM (12) provides a strong reference for general forecasting capacity, yet its performance indicates a gap between generic pretraining and surveillance-specific non-stationarity. EpiMap-LLM narrows this gap through lightweight, protocol-aware adaptation while keeping the backbone frozen.

**Table 1:** Main results on public-health forecasting benchmarks (JHU COVID-19 and CDC-IHA).

Family	Model	JHU COVID-19 (daily)		CDC-IHA (weekly)	
		RMSE ↓	MAE ↓	RMSE ↓	MAE ↓
Transformer	Informer (7)	0.382 ± 0.012	0.315 ± 0.009	6.842 ± 0.211	5.124 ± 0.155
	Autoformer (14)	0.375 ± 0.015	0.308 ± 0.011	7.155 ± 0.243	4.986 ± 0.142
Continuous-time	ContiFormer (11)	0.339 ± 0.008	0.282 ± 0.006	5.786 ± 0.185	4.341 ± 0.112
	ODE-RNN (9)	0.445 ± 0.021	0.347 ± 0.015	10.571 ± 0.432	9.504 ± 0.311
	Neural-CDE (10)	0.572 ± 0.025	0.528 ± 0.019	8.286 ± 0.364	7.104 ± 0.225
GNN-based	T-PatchGNN (15)	0.367 ± 0.018	0.305 ± 0.012	9.998 ± 0.311	8.372 ± 0.214
Foundation	TimesFM (12)	2.445 ± 0.150	0.338 ± 0.022	16.414 ± 1.102	4.628 ± 0.183
**Ours**	**EpiMap-LLM**	**0.289** **±0.005**	**0.241** **±0.004**	**4.918** **±0.122**	**3.732** **±0.086**

Lower is better. Bold denotes the best performance, and underline indicates the second best. Shaded cells (blue) highlight the best within each metric. Standard deviations (± std) are reported for five random seeds.

### 3.2 Additional generalization analyses

Tables 2, 3 assess the flexibility of EpiMap-LLM from two practical perspectives: (i) the choice of Temporal State Encoder (TSE) used to summarize surveillance trajectories, and (ii) the choice of the frozen LLM backbone used for downstream conditioning.

**Table 2:** Forecasting performance across different Temporal State Encoder (TSE) backbones.

TSE backbone	JHU COVID-19 (daily)		CDC-IHA (weekly)	
	RMSE ↓	MAE ↓	RMSE ↓	MAE ↓
GRU-TSE	0.368 ± 0.014	0.312 ± 0.009	7.210 ± 0.284	5.850 ± 0.192
Patch-TSE	0.342 ± 0.011	0.285 ± 0.007	6.150 ± 0.215	4.920 ± 0.144
ODE-TSE	0.315 ± 0.008	0.264 ± 0.005	5.420 ± 0.166	4.180 ± 0.105
Attn-TSE (ours)	**0.289** **±0.005**	**0.241** **±0.004**	**4.918** **±0.122**	**3.732** **±0.086**

Lower is better. Bold indicates the best performance, while underline indicates the second best.

**Table 3:** Performance comparison across different frozen LLM backbones with Attn-TSE.

LLM foundation	JHU COVID-19 (daily)		CDC-IHA (weekly)	
	RMSE ↓	MAE ↓	RMSE ↓	MAE ↓
Mistral-7B-v0.3	0.334 ± 0.012	0.288 ± 0.009	5.645 ± 0.194	4.412 ± 0.133
LLaMA-3-8B	0.301 ± 0.009	0.252 ± 0.006	5.120 ± 0.145	3.895 ± 0.098
Qwen-2.5-7B	**0.289** **±0.005**	**0.241** **±0.004**	**4.918** **±0.122**	**3.732** **±0.086**

Lower is better. Bold indicates the best performance.

Effect of the TSE backbone. Table 2 shows a consistent pattern across both benchmarks: stronger temporal state modeling translates into more effective conditioning and lower forecasting error. GRU-TSE is a competitive lightweight option, but it trails patch-based and continuous-time variants, suggesting that simple recurrent updates are often insufficient for surveillance streams where non-stationarity and reporting noise co-occur. Patch-TSE improves on GRU-TSE by capturing local temporal structure, while ODE-TSE further reduces error in settings with irregularity and revisions, consistent with the value of continuous-time dynamics when observations are shaped by reporting processes. Attn-TSE performs best on both JHU (daily) and CDC-IHA (weekly), indicating that attention-based state extraction provides the most stable interface for subsequent semantic conditioning, particularly when dependencies span multiple temporal scales and when reporting artifacts interact with epidemiological changes.

Effect of the frozen LLM backbone. Table 3 shows that EpiMap-LLM remains effective across different choices of frozen LLM backbones. All backbones use the same protocol-aware conditioning pipeline, and stronger backbones generally yield lower errors, with the clearest improvements on CDC-IHA where revisions and backfill amplify protocol effects. Importantly, the performance differences across LLMs are modest compared with what is typically observed under full fine-tuning. This suggests that the proposed adapters and anchors provide a stable, parameter-efficient interface for reusing frozen representations in surveillance forecasting, while reducing sensitivity to backbone choice in practical deployments.

### 3.3 Component contributions

Table 4 isolates the contribution of each core component. Two patterns stand out.

**Table 4:** Ablation study on core components (TSE, SPA, and PAB) in EpiMap-LLM.

Model variant	JHU COVID-19 (daily)		CDC-IHA (weekly)	
	RMSE ↓	MAE ↓	RMSE ↓	MAE ↓
EpiMap-LLM *w/o* TSE	0.785 ± 0.032	0.642 ± 0.024	12.450 ± 0.510	10.125 ± 0.385
EpiMap-LLM *w/o* SPA	0.355 ± 0.015	0.298 ± 0.010	6.220 ± 0.245	4.950 ± 0.166
EpiMap-LLM *w/o* PAB	0.315 ± 0.009	0.262 ± 0.007	6.550 ± 0.210	5.140 ± 0.152
EpiMap-LLM (Full)	**0.289** **±0.005**	**0.241** **±0.004**	**4.918** **±0.122**	**3.732** **±0.086**

Lower is better. Bold indicates the best performance. Standard deviations (± std) are reported for five random seeds.

Temporal state modeling is indispensable. Removing the Temporal State Encoder leads to a dramatic collapse on both benchmarks, indicating that the frozen LLM cannot compensate for missing temporal structure. This variant effectively discards the time-aligned latent epidemic trajectory, and the resulting prompts become too weak to support reliable multi-step forecasting.

SPA and PAB provide complementary gains, with PAB particularly important under protocol noise. Both *w/o SPA* and *w/o PAB* degrade performance relative to the full model, confirming that neither module is redundant. The SPA is necessary to translate latent epidemic states into a representation that the frozen LLM can use efficiently; without it, conditioning becomes a blunt projection and loses fidelity. The Protocol Anchor Bank yields additional improvements, and its impact is more pronounced on CDC-IHA, consistent with the stronger revision/backfill effects in weekly hospitalization surveillance. Together, these results support the central design choice of EpiMap-LLM: robust forecasting requires both a faithful state-to-LLM interface (SPA) and explicit protocol semantics (PAB), built on top of a competent temporal state backbone (TSE).

**Figure 2:**
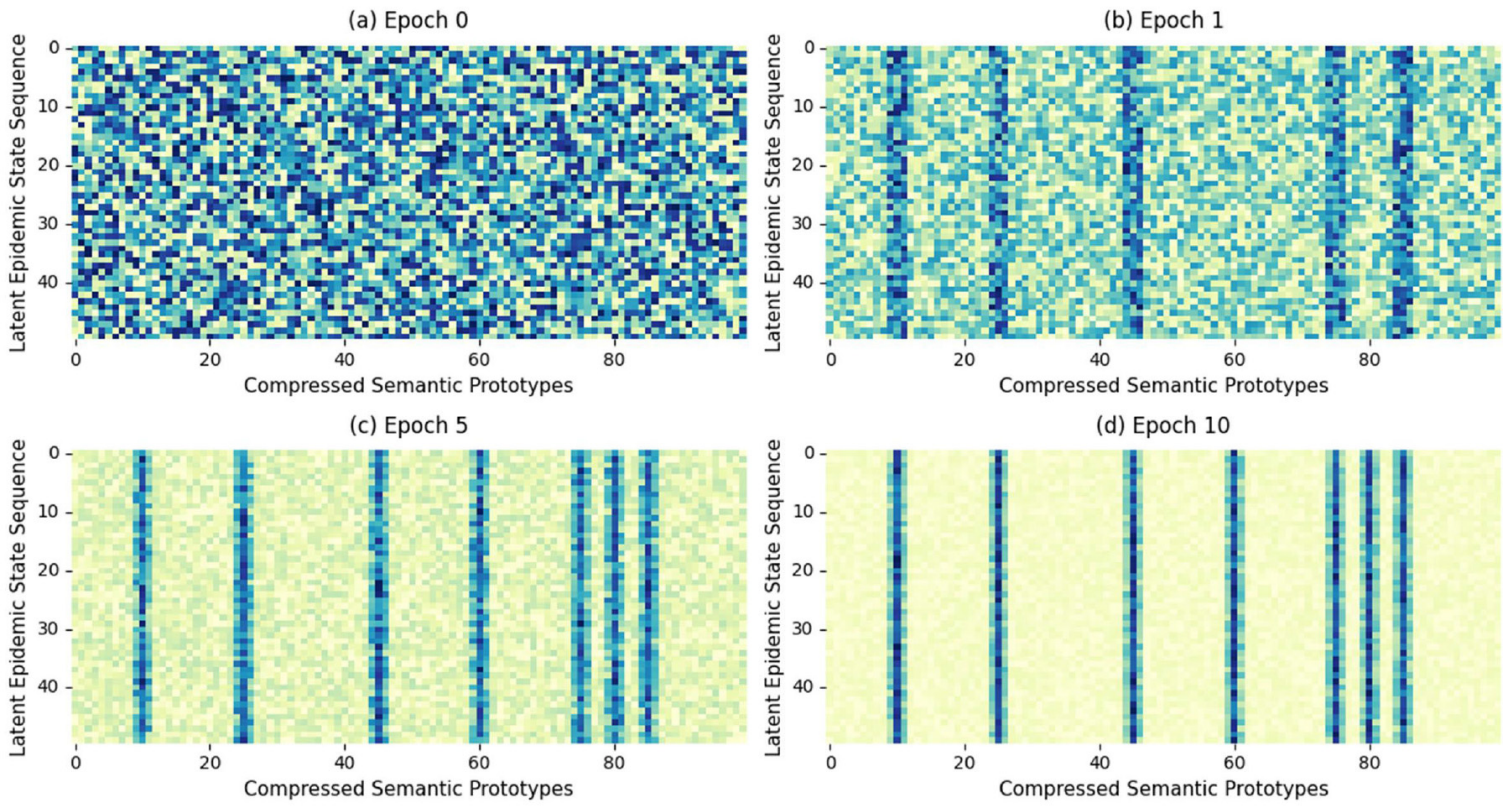
Alignment between latent epidemic states and compressed semantic prototypes across training epochs: **(a)** Epoch 0, **(b)** Epoch 1, **(c)** Epoch 5, and **(d)** Epoch 10. Brighter/darker vertical bands indicate semantic prototypes receiving concentrated attention from the epidemic state sequence.

Alignment dynamics. Figure 2 provides a qualitative view of how protocol-aware alignment emerges during training. At epoch 0, cross-attention is diffuse, suggesting that latent states have not yet discovered useful semantic support. As training proceeds, attention concentrates into stable vertical bands (epochs 1–10), indicating that many time steps consistently route through a small subset of compressed protocol prototypes. This progressive sparsification is consistent with the intended role of the adapter: it organizes noisy numerical trajectories into a structured, protocol-aware semantic space that the frozen LLM can reliably condition on.

### 3.4 Robustness under reporting irregularities

We study two deployment-critical robustness settings: (1) data-scarce regimes, which emulate the early stage of an outbreak where only a short history is available for training, and (2) cross-protocol transfer, which emulates training under one surveillance protocol (e.g., JHU daily reporting) and deploying under another (e.g., CDC weekly reporting) without additional fine-tuning.

Data-scarce regimes. Table 5 shows that EpiMap-LLM retains a clear advantage as the training ratio *p* decreases, with the largest gains in the most data-limited setting (*p* = 0.05). This pattern suggests that protocol-aware conditioning acts as a strong inductive bias when purely numerical models are forced to extrapolate from very short histories. Two mechanisms are likely at play: (i) the frozen LLM backbone provides a stable representation space that reduces the effective degrees of freedom during optimization, and (ii) the Protocol Anchor Bank supplies protocol-specific context that discourages fitting spurious fluctuations common in early-stage surveillance streams. Notably, the gap is more pronounced on CDC-IHA, consistent with the heavier reporting revisions/backfill in weekly hospitalization surveillance.

**Table 5:** Performance in data-scarce regimes (MAE/RMSE).

Dataset	Train ratio (*p*)	ContiFormer		EpiMap-LLM (ours)	
		MAE ↓	RMSE ↓	MAE ↓	RMSE ↓
JHU COVID-19 (Daily)	0.05	0.452 ± 0.022	0.584 ± 0.035	**0.312** **±0.009**	**0.385** **±0.012**
	0.10	0.388 ± 0.015	0.492 ± 0.021	**0.285** **±0.007**	**0.344** **±0.009**
	0.20	0.322 ± 0.011	0.415 ± 0.018	**0.264** **±0.005**	**0.318** **±0.006**
CDC-IHA (Weekly)	0.05	8.520 ± 0.412	11.240 ± 0.650	**4.850** **±0.155**	**6.240** **±0.211**
	0.10	6.440 ± 0.243	8.986 ± 0.322	**4.225** **±0.110**	**5.633** **±0.164**
	0.20	5.120 ± 0.185	6.842 ± 0.215	**3.984** **±0.092**	**5.210** **±0.125**

Each model is trained on a fraction *p* of the training timeline and evaluated on the same held-out test window. Lower is better. Bold values indicate the best performance for each metric under the same dataset and train ratio.

Cross-protocol transfer. Table 6 evaluates robustness under severe protocol shifts in cadence and revision behavior. Patch-TSE deteriorates substantially when transferring across regimes, indicating limited invariance to changes in temporal aggregation and reporting artifacts. In contrast, EpiMap-LLM degrades more gracefully in both directions. This supports the intended role of the Protocol Anchor Bank: it exposes the reporting regime as an explicit conditioning factor, allowing the model to reinterpret latent epidemic states under a new protocol rather than treating the shift as a change in the underlying disease dynamics. Overall, these results suggest that protocol-aware semantic conditioning is a practical mechanism for deployment across heterogeneous surveillance systems.

**Table 6:** Cross-protocol generalization results.

Source regime	Target regime	Patch-TSE		EpiMap-LLM	
		MAE ↓	RMSE ↓	MAE ↓	RMSE ↓
JHU (Daily)	CDC (Weekly)	9.452	12.840	**5.315**	**6.984**
CDC (Weekly)	JHU (Daily)	0.612	0.835	**0.388**	**0.492**

Models are trained on a source surveillance regime and directly evaluated on a target regime without target-domain fine-tuning. Lower is better. Bold indicates the best performance.

### 3.5 Operational reliability and surge detection

While global error metrics (MAE/RMSE) evaluate point-forecast accuracy, real-world public health information systems primarily rely on forecasting models to trigger early warnings for capacity planning (e.g., ICU bed allocation). To evaluate operational reliability, we translate the continuous forecasting outputs into a decision-relevant binary classification task: *Surge Detection*.

Setup. We define a “surge” as a relative increase of ≥20% in hospitalizations over a 2-week forward window. We evaluate this on the weekly CDC-IHA dataset, which is notoriously affected by reporting delays. To strictly emulate real-time operational constraints, models are forced to make predictions using *provisional data* (i.e., the initially reported, incomplete counts before retrospective backfill is applied). We report Precision, Recall (Sensitivity), and the F1-score for surge detection. We compare EpiMap-LLM against the strongest deep learning baseline (ContiFormer) and a standard operational baseline (ARIMA) (16).

Table 7 illustrates a critical failure mode of conventional forecasting architectures when deployed in live health information systems. When fed with provisional data, standard numerical models like ContiFormer exhibit poor Recall (0.482). Because recent weeks' data are artificially suppressed by reporting delays, purely data-driven models interpret this administrative artifact as a genuine decline in transmission. Consequently, they systematically miss impending surges (producing false negatives), which in a clinical setting could lead to delayed resource mobilization.

**Table 7:** Operational reliability for early surge detection using real-time provisional CDC-IHA data.

Model	Precision ↑	Recall ↑	F1-score ↑
ARIMA (Operational Baseline)	0.412	0.355	0.381
ContiFormer (Best DL Baseline)	0.534	0.482	0.506
**EpiMap-LLM (ours)**	**0.685**	**0.724**	**0.703**

A surge is defined as a ≥20% increase over a 2-week horizon. Bold indicates best performance.

Classical statistical methods like ARIMA struggle similarly, often lagging behind the actual epidemiological curve and producing low precision due to over-extrapolating past trends.

In contrast, EpiMap-LLM achieves a substantially higher Recall (0.724) and F1-score (0.703). This stability is a direct consequence of the Protocol Anchor Bank. By explicitly conditioning the LLM on a “provisional/backfill” semantic anchor, By explicitly conditioning the model on a “provisional/backfill” semantic anchor, the framework helps disentangle reporting-delay effects from the underlying disease momentum at the representation level. The LLM learns to calibrate the artificially low recent counts against its pre-trained understanding of epidemic growth phases. As a result, EpiMap-LLM successfully anticipates upward trajectories even when the raw, unadjusted inputs misleadingly suggest a downward trend. This diagnostic capability bridges the gap between raw data processing and actionable public health intelligence, making the model highly reliable for deployment in automated surveillance dashboards.

### 3.6 Expert evaluation and interpretability

A persistent barrier to deploying predictive models in health information systems is limited user trust in black-box outputs, particularly when model predictions conflict with provisional surveillance data that may be delayed, incomplete, or later revised. EpiMap-LLM uses a frozen generative backbone, which allows the same protocol-aware prompt embeddings used for forecasting to also support concise natural-language rationales, enabling a consistent interface between numerical predictions and practitioner-facing explanations.

Evaluation setup. We conducted an expert evaluation with 12 public health practitioners (e.g., epidemiologists and hospital capacity managers). Each evaluator reviewed 30 retrospective forecasting scenarios from the CDC-IHA dataset, selected to emphasize periods with pronounced reporting anomalies (e.g., holiday-related reporting disruptions and subsequent backfill). For each scenario, evaluators were shown the raw provisional observations available at the forecast time together with forecasts from three systems: (i) ARIMA as an operational statistical baseline, (ii) ContiFormer as a representative deep learning forecaster, and (iii) EpiMap-LLM. For EpiMap-LLM, we additionally generated a short, standardized two-sentence rationale conditioned on the same protocol-aware tokens used for prediction (e.g., “*The recent decrease is consistent with a reporting cadence disruption; revision patterns suggest the underlying trajectory remains upward.”*). Scenarios and systems were presented in randomized order, and evaluators were blinded to model identity.

Measures. Evaluators rated each system on a 5-point Likert scale along three dimensions relevant to decision support: *Diagnostic Clarity* (whether the output helps interpret apparent anomalies in the surveillance stream), *Actionability* (confidence to make operational adjustments based on the forecast), and *Overall Trust* (global confidence in the system under reporting rregularities).

Findings. Table 8 shows that explanation-supported outputs materially improved practitioner-facing utility during revision-heavy periods. Although the deep forecaster provided reasonable numerical accuracy, evaluators rated its diagnostic clarity and trust relatively low, noting that unexpected divergences from the provisional trajectory were difficult to interpret without an explicit account of reporting artifacts.

**Table 8:** Expert evaluation on a 5-point Likert scale (mean ± standard deviation) during anomalous reporting periods (*N* = 12 evaluators, 30 scenarios). Bold indicates the highest rating.

Forecasting system	Diagnostic clarity	Actionability	Overall trust
ARIMA (Statistical Baseline)	2.15 ± 0.62	2.40 ± 0.55	2.35 ± 0.61
ContiFormer (Deep Forecaster)	2.55 ± 0.81	2.75 ± 0.72	2.60 ± 0.78
**EpiMap-LLM (w/ Rationale)**	**4.35** ±**0.48**	**4.10** ±**0.52**	**4.25** ±**0.45**

In contrast, EpiMap-LLM received substantially higher ratings across all three dimensions. Evaluators reported that brief rationales referencing reporting cadence and revision patterns helped reconcile discrepancies between raw provisional observations and the forecast, and increased confidence when making operational judgments under uncertainty. These results suggest that coupling protocol-aware forecasting with concise, standardized explanations can improve human-in-the-loop usability, making EpiMap-LLM more suitable as a forecasting component in practitioner-facing surveillance dashboards.

## 4 Discussion

Public-health forecasting is a core component of epidemic intelligence, early warning, and operational decision support. In real deployment, surveillance time series are shaped not only by disease dynamics but also by reporting pipelines—including right-truncation, revisions, and backfill—that can destabilize model behavior across regions and systems. Our results show that explicitly modeling these protocol semantics is practically valuable: by linking numerical epidemic dynamics with protocol-aware conditioning, EpiMap-LLM delivers stronger and more stable forecasts under heterogeneous surveillance conditions. We emphasize that the present study is positioned as a protocol-aware forecasting framework, rather than as a dedicated nowcasting benchmark against the full family of revision-aware public-health methods.

The gains on CDC-IHA are especially important because this dataset reflects revision-heavy weekly surveillance used in dashboard monitoring and near-term hospital planning. Better robustness in this setting suggests more reliable forecasts when provisional reports are incomplete and later updated. In practice, this can support more dependable surge tracking and earlier response planning.

The expert evaluation also suggests better diagnostic clarity and user trust when short protocol-aware rationales accompany forecasts. However, this remains preliminary evidence rather than definitive validation, and larger prospective studies in real surveillance workflows are still needed.

## 5 Limitations and future work

This study has several limitations. First, our evaluation focuses on two widely used surveillance benchmarks; additional validation on other diseases, jurisdictions, and data sources (e.g., syndromic surveillance and wastewater signals) is needed to assess broader generality. Second, we primarily report point-forecast metrics, and while the framework is compatible with probabilistic forecasting, we did not fully evaluate calibration and uncertainty quality under the standard probabilistic scoring rules used in some collaborative forecasting settings. Third, although the paper focuses on forecasting robustness under heterogeneous reporting systems, our comparison set is not intended to cover the full family of revision-aware or nowcasting-specific public-health methods; such comparisons are outside the present empirical scope and should be interpreted as an important direction for future work. Fourth, our protocol meta-data and anchoring design capture common reporting properties, but surveillance systems differ in their revision policies and data definitions; performance may depend on the availability and accuracy of such meta-data. Finally, the expert evaluation was conducted at a modest scale and should be interpreted as an initial assessment of usability rather than a definitive clinical or operational study.

Future work. Several directions follow naturally. An important next step is to evaluate the framework more directly against revision-aware and nowcasting-oriented public-health methods under task-aligned protocols. An immediate extension is to incorporate probabilistic forecasting and calibration analysis under revision-aware evaluation protocols, enabling closer alignment with operational risk thresholds.

Another direction is to broaden the protocol anchor set to cover richer, system-specific semantics (e.g., changes in case definitions, reporting policy updates, and cross-source reconciliation) and to study automated detection of protocol changes from data streams. In addition, larger and more diverse human-in-the-loop studies, including prospective evaluations embedded in real surveillance dashboards, would help clarify how explanation style, timing, and presentation affect decision-making. Finally, integrating EpiMap-LLM with multi-source surveillance pipelines (e.g., combining clinical, laboratory, and digital signals) may further improve robustness in settings where any single stream is incomplete or delayed.

## 6 Conclusion

EpiMap-LLM provides a protocol-aware forecasting interface that links epidemic dynamics with surveillance semantics under heterogeneous reporting systems. Across daily and weekly benchmarks, the framework improves point-forecast accuracy and stability under revision-heavy conditions while preserving a lightweight adaptation strategy around a frozen backbone. These properties support practical use in surveillance dashboards and operational public-health decision support.

## Data Availability

Publicly available datasets were analyzed in this study. This data can be found here: JHU CSSE COVID-19 dataset (Johns Hopkins University Center for Systems Science and Engineering repository): https://github.com/CSSEGISandData/COVID-19 CDC influenza hospitalization surveillance (CDC FluSight forecasting page): https://www.cdc.gov/flu/weekly/flusight/flu-forecasting.html.
