# Supplementary material for: Protocol-aware epidemic forecasting across heterogeneous public health surveillance systems
Source: Front Public Health. 2026 May 29;14:1829302. doi: 10.3389/fpubh.2026.1829302 (PMC13260628; doi:10.3389/fpubh.2026.1829302)
Supplement: Supplementary file 1 [file Data_Sheet_1.pdf]

frontiers

Appendix to the Main Manuscript: Additional Methods, Baselines, and Experimental Details

1 APPENDIX OVERVIEW

This document serves as the **supplementary appendix** to the main manuscript. It provides additional technical and experimental details that complement, but are not repeated in full in, the main text.

Specifically, this appendix is organized as follows:

- **Appendix A (Related Works):** expanded background context for epidemic forecasting and LLM-based time-series modeling.
- **Appendix B (Methods):** full formulation, architecture details, training objective, and implementation settings corresponding to the main-text Methods section.
- **Appendix C (Experiments):** dataset protocol, baseline setup, and extended experimental details supporting the main-text results.

2 APPENDIX A: RELATED WORKS

2.1 Epidemic Time-Series Forecasting

Public-health forecasting has historically relied on mechanistic compartmental models and classical statistical approaches (e.g., ARIMA and Bayesian hierarchical models) to anticipate disease trajectories McGowan et al. (2019); Reich et al. (2019). While highly interpretable, these methods often struggle to capture non-linear, high-dimensional temporal dependencies during rapidly evolving outbreaks. The COVID-19 pandemic accelerated the adoption of deep learning in epidemiology, driving the creation of large-scale collaborative forecasting infrastructures, such as the US COVID-19 Forecast Hub and the JHU CSSE dashboards Cramer et al. (2022); Dong et al. (2022); Ray et al. (2023). Concurrently, some works have demonstrated the efficacy of hybrid machine learning frameworks in capturing complex epidemic curves Zivkovic et al. (2021); Zeroual et al. (2020). In public-health practice, collaborative forecasting systems also emphasize standardized targets and evaluation protocols; while many settings support probabilistic submissions, reliable point forecasts remain a common operational input, especially when reporting artifacts and protocol shifts dominate real-time uncertainty.

In the broader machine learning community, time-series forecasting has been advanced by Transformer architectures. Models such as Informer Zhou et al. (2021), Autoformer Wu et al. (2021), and PatchTST Nie et al. (2023) have shown strong performance on regular, long-horizon forecasting tasks. Recently, this paradigm has scaled into zero-shot foundation models, such as TimesFM Das et al. (2024), which pre-train massive decoders on extensive cross-domain corpora to generalize across diverse temporal domains.

However, public-health surveillance streams are notoriously irregular, characterized by reporting delays, right-truncation, and retrospective backfill Höhle and an der Heiden (2014). From a health information perspective, these streams are often versioned: preliminary releases are revised as additional reports arrive and validation processes complete, which complicates supervision, auditability, and comparability across jurisdictions and surveillance systems. To address temporal irregularity, specialized architectures have emerged. Continuous-time models—such as ODE-RNN Rubanova et al. (2019) and Neural-CDE

Kidger et al. (2020)—treat observations as samples from underlying continuous dynamics, a concept recently extended by the continuous-time transformer ContiFormer Chen et al. (2023). Concurrently, graph-based approaches like T-PatchGNN Zhang et al. (2024) dynamically align variable-length patches to capture complex inter-series correlations across asynchronous observations. Despite these architectural advances, existing deep models overwhelmingly treat surveillance data as purely numerical vectors. They implicitly assume that observation gaps or sudden spikes are natural phenomena, failing to recognize them as artifacts of the reporting protocol and revision lifecycle. Consequently, they often suffer severe performance degradation under cross-region distribution shifts and cross-system protocol changes. These limitations motivate forecasting approaches that explicitly encode reporting semantics while preserving the capacity of temporal representation learning; our work follows this direction by linking latent epidemic dynamics with protocol-aware conditioning.

2.2 Large Language Models for Time-Series Modeling

The reasoning and few-shot generalization capabilities of large language models (LLMs) have recently motivated their adaptation for time-series tasks. A dominant paradigm involves treating time-series forecasting as a generative modeling problem. Foundation models such as TimesFM Das et al. (2024) and Chronos Ansari et al. (2024) pre-train massive transformer decoders on extensive cross-domain time-series corpora, demonstrating strong zero-shot capabilities.

Alternatively, researchers have attempted to adapt pre-trained natural language LLMs (e.g., LLaMA, GPT) directly to numerical sequences. Methods like PromptCast Xue and Salim (2023) and LLM4TS Chang et al. (2024) serialize numerical values into text strings to leverage the native inference behavior of the language model. Furthermore, Time-LLM Jin et al. (2023) and UniTS Gao et al. (2024) employ reprogramming techniques to align time-series patches with textual embedding spaces. While promising, directly serializing numerical histories into textual templates introduces critical bottlenecks: it rapidly exhausts the LLM context window, reduces numerical fidelity, and can yield unreliable outputs when the numerical distribution deviates from the text-heavy pre-training corpus, which is also difficult to standardize and audit in surveillance pipelines. In contrast, our proposed *Semantic Prompt Adapter* avoids brittle full-history text serialization. By projecting latent epidemic states directly into continuous prompt embeddings, EpiMap-LLM enables a frozen LLM to condition on dense numerical dynamics within a protocol-aware semantic interface efficiently.

3 APPENDIX B: METHODS (DETAILED VERSION OF MAIN-TEXT METHODS)

3.1 Problem Formulation and Clarification of Protocol-aware Decoupling

To address reporting revisions and heterogeneous surveillance pipelines, we formulate epidemic forecasting from surveillance streams augmented with protocol metadata. Let $\mathbf{x}_t \in \mathbb{R}^{d_x}$ denote the multivariate observation vector at time t (e.g., cases, hospitalizations, deaths, and auxiliary indicators), and let $\mathbf{m}_t$ denote reporting meta-data that characterize the surveillance pipeline (e.g., reporting cadence, spatial aggregation level, and revision/backfill status). For each location (or surveillance series), we observe a length-T sequence

$$\mathcal{S} = \{(\mathbf{x}_1, \mathbf{m}_1), \dots, (\mathbf{x}_T, \mathbf{m}_T)\}. \tag{S1}$$

We focus on multi-horizon forecasting with $h \in \{1, \dots, H\}$, where the target is $y_{t+h} \in \mathbb{R}$ (or $\mathbf{y}_{t+h} \in \mathbb{R}^{d_y}$ for multivariate targets). To reflect right-truncation and retrospective revisions in real-time surveillance, we use a validity mask $v_{t+h} \in \{0, 1\}$ to indicate whether y_{t+h} is available and suitable for supervision at

training time (with $v_{t+h} = 0$ covering missing or not-yet-finalized targets). To make the role of reporting artifacts explicit, we further view the observed surveillance value as being jointly shaped by two sources of variation: the underlying epidemic dynamics and the reporting process. A simple conceptual formulation is

$$\mathbf{x}_t^{(r)} = g(\mathbf{z}_t, \mathbf{m}_t, r) + \boldsymbol{\epsilon}_t, \tag{S2}$$

where $\mathbf{z}_t$ denotes the latent epidemic state, $\mathbf{m}_t$ denotes protocol/reporting metadata, r denotes the reporting version or revision state, and $\mathbf{x}_t^{(r)}$ is the corresponding observed value at time t. Under this view, the surveillance stream should not be interpreted as a direct readout of epidemic dynamics alone, because cadence, aggregation rules, right-truncation, revisions, and backfill may all distort the provisional signal seen by a forecasting model. In this work, we therefore use the term protocol-aware decoupling in a representation-level and operational sense, rather than as a claim of exact causal recovery of an unobserved epidemic truth. More specifically, decoupling refers to learning a structured forecasting interface in which information about the epidemic trajectory is encoded through temporal state representations, while protocol-related variation is exposed through an explicit protocol-conditioning pathway. Operationally, this means encouraging the model to be less sensitive to administrative perturbations that do not correspond to genuine epidemiological changes, while remaining responsive to signal components that are predictive of future disease burden.

Under this definition, the objective of EpiMap-LLM is not to perform a strict causal deconvolution of the surveillance process. Instead, the model aims to preserve predictive information about the latent epidemic trajectory while making reporting-process effects explicit and controllable during forecasting. This interpretation directly motivates the two-pathway design described below: the Temporal State Encoder captures trajectory-level epidemic information, whereas the Protocol Anchor Bank represents reporting semantics explicitly.

4 APPENDIX B: METHODS (DETAILED VERSION OF MAIN-TEXT METHODS)

4.1 Problem Formulation and Clarification of Protocol-aware Decoupling

To address reporting revisions and heterogeneous surveillance pipelines, we formulate epidemic forecasting from surveillance streams augmented with protocol metadata. Let $\mathbf{x}_t \in \mathbb{R}^{d_x}$ denote the multivariate observation vector at time t (e.g., cases, hospitalizations, deaths, and auxiliary indicators), and let $\mathbf{m}_t$ denote reporting meta-data that characterize the surveillance pipeline (e.g., reporting cadence, spatial aggregation level, and revision/backfill status). For each location (or surveillance series), we observe a length-T sequence

$$\mathcal{S} = \{(\mathbf{x}_1, \mathbf{m}_1), \ldots, (\mathbf{x}_T, \mathbf{m}_T)\}. \tag{S3}$$

We focus on multi-horizon forecasting with $h \in \{1, \ldots, H\}$, where the target is $y_{t+h} \in \mathbb{R}$ (or $\mathbf{y}_{t+h} \in \mathbb{R}^{d_y}$ for multivariate targets). To reflect right-truncation and retrospective revisions in real-time surveillance, we use a validity mask $v_{t+h} \in \{0, 1\}$ to indicate whether y_{t+h} is available and suitable for supervision at training time (with $v_{t+h} = 0$ covering missing or not-yet-finalized targets).

To make the role of reporting artifacts explicit, we further view the observed surveillance value as being jointly shaped by two sources of variation: the underlying epidemic dynamics and the reporting process. A simple conceptual formulation is

$$\mathbf{x}_t^{(r)} = g(\mathbf{z}_t, \mathbf{m}_t, r) + \boldsymbol{\epsilon}_t, \tag{S4}$$

where $\mathbf{z}_t$ denotes the latent epidemic state, $\mathbf{m}_t$ denotes protocol/reporting metadata, r denotes the reporting version or revision state, and $\mathbf{x}_t^{(r)}$ is the corresponding observed value at time t. Under this view, the surveillance stream should not be interpreted as a direct readout of epidemic dynamics alone, because cadence, aggregation rules, right-truncation, revisions, and backfill may all distort the provisional signal seen by a forecasting model.

In this work, we therefore use the term protocol-aware decoupling in a representation-level and operational sense, rather than as a claim of exact causal recovery of an unobserved epidemic truth. More specifically, decoupling refers to learning a structured forecasting interface in which information about the epidemic trajectory is encoded through temporal state representations, while protocol-related variation is exposed through an explicit protocol-conditioning pathway. Operationally, this means encouraging the model to be less sensitive to administrative perturbations that do not correspond to genuine epidemiological changes, while remaining responsive to signal components that are predictive of future disease burden.

Under this definition, the objective of EpiMap-LLM is not to perform a strict causal deconvolution of the surveillance process. Instead, the model aims to preserve predictive information about the latent epidemic trajectory while making reporting-process effects explicit and controllable during forecasting. This interpretation directly motivates the hybrid design described below: a lightweight temporal encoder first captures trajectory-level epidemic information, dedicated protocol-aware modules then expose reporting semantics explicitly, and a frozen LLM backbone finally integrates these aligned representations for forecasting.

4.2 Overall Architecture

To preserve temporal information and avoid brittle text serialization, EpiMap-LLM is designed as a hybrid architecture rather than either a pure time-series predictor or a text-based LLM forecaster in the usual sense. Its key idea is to combine: (i) a lightweight temporal modeling component for extracting epidemic dynamics from raw surveillance trajectories, (ii) lightweight alignment and protocol-aware modules that translate task-specific numerical information into the LLM space, and (iii) a frozen LLM backbone that serves as a semantic integration layer over these heterogeneous representations. To preserve temporal information and avoid brittle text serialization, EpiMap-LLM treats forecasting as a protocol-aware interface problem. The model separates epidemiological dynamics from reporting artifacts rather than treating protocol effects as generic noise. As illustrated in Fig. S1, EpiMap-LLM includes three trainable modules that interface with the frozen backbone: (i) a **Temporal State Encoder** that compresses multivariate trajectories into time-aligned latent epidemic state tokens, thereby performing the primary task-specific numerical modeling of epidemic dynamics; (ii) a **State Prompt Adapter (SPA)** that maps latent states into continuous prompt embeddings compatible with the frozen embedding space, thereby acting as a semantic alignment module between the temporal model and the frozen LLM; and (iii) a **Protocol Anchor Bank** that produces protocol-aware tokens encoding reporting semantics (e.g., cadence, spatial granularity, and revision/backfill patterns), thereby making the reporting context explicitly available within the same LLM-compatible representation space. In addition, we construct a short, fixed **Context Header** from static context features (e.g., recent growth and seasonality proxies) and embed it through the frozen input embedding layer, yielding **Context Header Tokens**. The frozen backbone then integrates these token types through its pretrained embedding and attention space. Accordingly, the role of the LLM in our framework is not conventional text prompting or explicit natural-language reasoning, but high-level semantic integration over continuous prompt embeddings and protocol-aware tokens. The

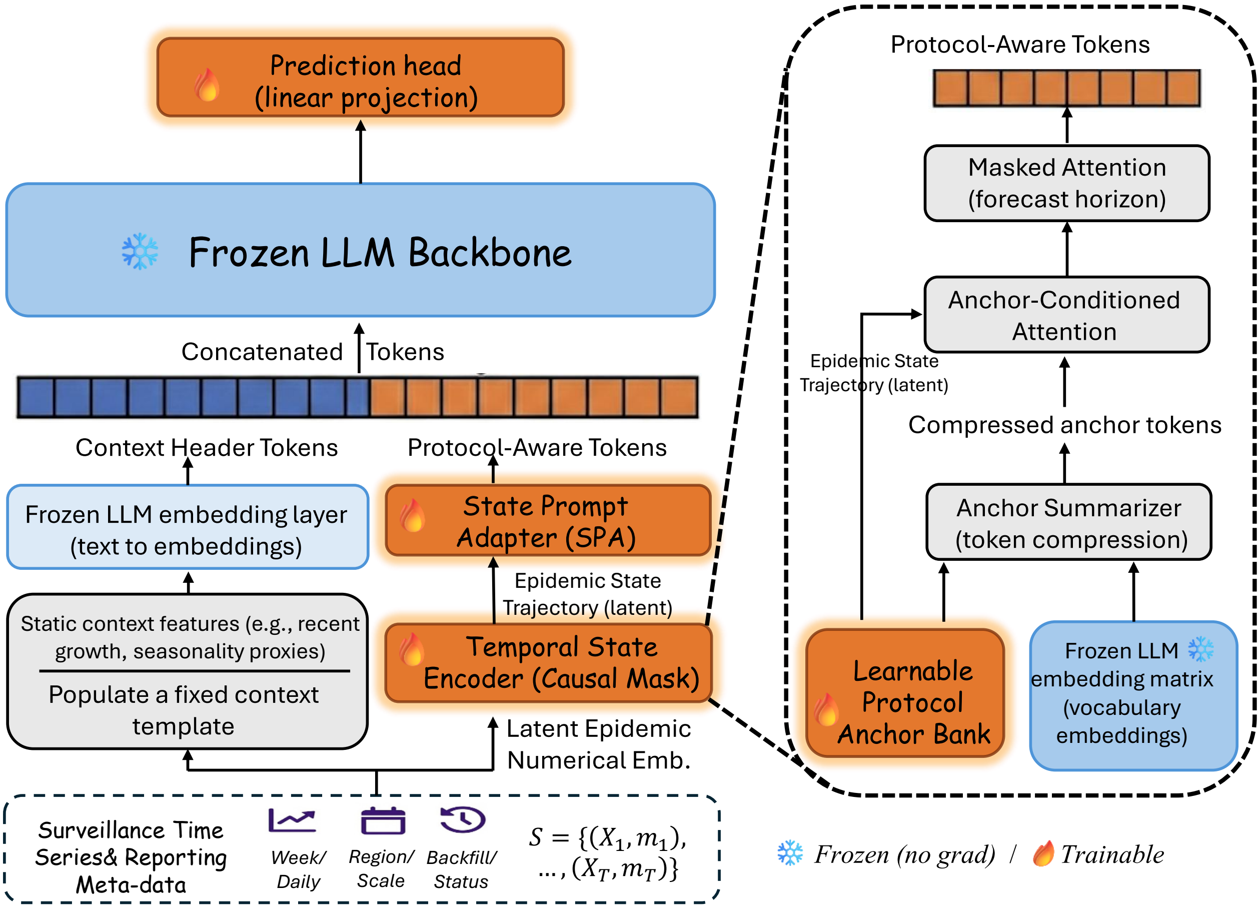

Figure S1: Overview of **EpiMap-LLM**. A temporal state encoder extracts latent epidemic states from surveillance trajectories; a state prompt adapter and protocol anchor bank inject state and reporting semantics into a frozen LLM for forecasting. The overall framework is hybrid: the temporal encoder captures task-specific epidemic dynamics, the adapter and protocol-aware modules align these signals to the frozen LLM space, and the frozen LLM serves as the semantic integration backbone. Snowflake/flame denote frozen/trainable modules.

frozen backbone integrates the concatenated tokens via self-attention, and a lightweight **Prediction Head** produces multi-horizon forecasts.

4.3 Temporal State Encoder

To preserve high-frequency surveillance dynamics, we encode surveillance streams as compact latent state trajectories instead of textual histories. Directly converting a long history into text is context-inefficient and sensitive to formatting, while also blurring high-frequency temporal signals. We therefore encode the sequence into a compact latent state trajectory.

Shifted forecasting events. To predict future targets using only observed prefixes, we construct shifted composite inputs:

$$\mathbf{e}_t = \phi(\mathbf{x}_t, \mathbf{m}_t) \in \mathbb{R}^{d_e}, \quad t = 1, \ldots, T, \tag{S5}$$

where $\phi(\cdot)$ is a learnable embedding function for numerical observations and reporting meta-data. Stacking all embeddings yields $\mathbf{E} = [\mathbf{e}_1, \ldots, \mathbf{e}_T] \in \mathbb{R}^{T \times d_e}$.

Causal temporal encoding. The Temporal State Encoder aggregates $\mathbf{E}$ into a sequence of latent epidemic state tokens:

$$\mathbf{H} = f_{\text{temp}}(\mathbf{E}) \in \mathbb{R}^{T \times d_h}, \tag{S6}$$

where f_{temp} can be instantiated by different time-series backbones. In this work we use a causal-masked self-attention encoder so that each $\mathbf{h}_t$ depends only on $\{(\mathbf{x}_i, \mathbf{m}_i)\}_{i \le t}$, preventing leakage from future observations. This module should therefore be understood as the task-specific temporal modeling component of the framework: it extracts trajectory-level epidemic information before any interaction with the frozen LLM occurs.

4.4 State Prompt Adapter

To align task-specific epidemic states with a frozen backbone, we map latent states into the LLM embedding space through a lightweight adapter. The **State Prompt Adapter (SPA)** is not merely a generic linear projection. Its role is to act as a semantic alignment layer that maps latent epidemic states into continuous prompt embeddings compatible with the frozen LLM space. This design allows the model to reuse the pretrained representational structure of the LLM without serializing long numerical histories into brittle text prompts. The **State Prompt Adapter (SPA)** provides a parameter-efficient interface that maps latent states into continuous prompt embeddings compatible with the frozen LLM.

We project each latent state to the LLM embedding dimension D:

$$\mathbf{P}_{\text{state}} = \mathbf{H}\mathbf{W}_s \in \mathbb{R}^{T \times D}, \tag{S7}$$

where $\mathbf{W}_s \in \mathbb{R}^{d_h \times D}$ is trainable. $\mathbf{P}_{\text{state}}$ serves as the state-conditioned soft prompt sequence injected via `inputs_embeds`. In this sense, the term adapter is intentional: the module does not replace the LLM, but makes time-series state representations legible to the frozen LLM backbone.

4.5 Protocol Anchor Bank

To expose surveillance protocol semantics explicitly, we model cadence, spatial aggregation, and revision/backfill patterns through a dedicated protocol anchor bank. Importantly, the **Protocol Anchor Bank (PAB)** should not be viewed as a separate alternative to the LLM. Rather, it is a protocol-aware adaptation mechanism designed to inject reporting semantics into the same frozen LLM representation space used by the state prompts. Thus, the protocol-aware components and the frozen LLM backbone are complementary parts of a single framework, not competing explanations for the final performance. EpiMap-LLM encodes these semantics through a **Protocol Anchor Bank** and injects **Protocol-Aware Tokens** as context/constraints.

Anchor bank and token compression. Let $\mathbf{W} \in \mathbb{R}^{|\mathcal{V}| \times D}$ be the frozen input embedding matrix of the LLM. The use of the frozen LLM embedding matrix here is deliberate: protocol anchors are constructed relative to the semantic basis of the frozen LLM, so that reporting semantics and epidemic-state semantics can later interact in a shared pretrained space. We maintain a learnable protocol anchor bank $\mathbf{A} \in \mathbb{R}^{N_a \times D}$ and summarize it into a compact set of anchor tokens to control computation. We use a learnable query bank $\mathbf{U} \in \mathbb{R}^{N' \times D}$ with $N' \ll N_a$ to compress anchors:

$$\mathbf{C} = \text{MHA}(\mathbf{U}, \mathbf{A}, \mathbf{A}) \in \mathbb{R}^{N' \times D}, \tag{S8}$$

where only $\mathbf{U}$ and $\mathbf{A}$ are trainable. This **Anchor Summarizer** produces a reusable compressed representation $\mathbf{C}$.

Anchor-conditioned attention. Given compressed anchor tokens $\mathbf{C}$ and latent state prompts $\mathbf{P}_{\text{state}}$, we form protocol-aware tokens by cross-attention:

$$\tilde{\mathbf{P}}_{\text{prot}} = \mathrm{MHA}(\mathbf{P}_{\text{state}}\mathbf{W}_Q,\ \mathbf{C}\mathbf{W}_K,\ \mathbf{C}\mathbf{W}_V) \in \mathbb{R}^{T\times D}, \tag{S9}$$

where $\mathbf{W}_Q \in \mathbb{R}^{D\times D}$, $\mathbf{W}_K, \mathbf{W}_V \in \mathbb{R}^{D\times D}$ are trainable. We then apply a masked self-attention refinement (forecast-horizon masking) over $\tilde{\mathbf{P}}_{\text{prot}}$:

$$\mathbf{P}_{\text{prot}} = \mathrm{MaskedSA}(\tilde{\mathbf{P}}_{\text{prot}}) \in \mathbb{R}^{T\times D}. \tag{S10}$$

$\mathbf{P}_{\text{prot}}$ are the final **Protocol-Aware Tokens**, encouraging the model to attribute fluctuations to reporting mechanisms versus genuine epidemiological shifts. These protocol-aware tokens are therefore not standalone predictors; rather, they are semantic carriers that make the reporting regime explicit before joint integration by the frozen LLM.

4.6 Frozen LLM Conditioning and Prediction

Context header tokens. We build a short fixed context header from static context features (e.g., recent growth and seasonality proxies), and embed it through the frozen LLM embedding layer to obtain:

$$\mathbf{P}_{\text{ctx}} \in \mathbb{R}^{L_{\text{ctx}}\times D}. \tag{S11}$$

This header provides stable, global context without encoding any future target information.

Hybrid input and frozen inference. At this stage, the frozen LLM backbone serves as the semantic integration layer of the overall framework. It receives context-header tokens, epidemic-state prompts, and protocol-aware semantic tokens in a shared pretrained embedding space, and models their interaction through its frozen self-attention stack. Accordingly, the benefit of the LLM in our setting does not come from natural-language input formatting or explicit text generation. Instead, it comes from providing a large pretrained semantic interaction space in which epidemic-state signals can be interpreted jointly with reporting-protocol information. We concatenate context header tokens and protocol-aware tokens (which already incorporate state prompts through anchor-conditioned attention):

$$\mathbf{X}_{\text{in}} = [\mathbf{P}_{\text{ctx}}; \mathbf{P}_{\text{prot}}] \in \mathbb{R}^{(L_{\text{ctx}}+T)\times D}, \tag{S12}$$

and feed $\mathbf{X}_{\text{in}}$ to the frozen LLM backbone via `inputs_embeds`:

$$\mathbf{O} = \mathrm{LLM}_{\text{frozen}}(\mathbf{X}_{\text{in}}) \in \mathbb{R}^{(L_{\text{ctx}}+T)\times D}. \tag{S13}$$

We select the last T hidden states corresponding to time-aligned positions:

$$\mathbf{O}_{\text{time}} = \mathbf{O}[-T:] \in \mathbb{R}^{T\times D}. \tag{S14}$$

Prediction head. A lightweight projection head outputs horizon-h forecasts:

$$\hat{y}_{t+h} = \mathbf{o}_t^\top \mathbf{w}_h + b_h, \quad \mathbf{o}_t \in \mathbb{R}^D, \tag{S15}$$

where $\{\mathbf{w}_h, b_h\}_{h=1}^{H}$ are trainable parameters and $\mathbf{o}_t$ is the t-th row of $\mathbf{O}_{\text{time}}$. In this way, the final forecast is produced from a representation that has already integrated epidemic dynamics and reporting semantics within the frozen LLM space.

4.7 Training Objective and Optimization

We train EpiMap-LLM with a masked multi-horizon objective that is robust to reporting artifacts. Specifically, we adopt a Huber loss with (i) backfill-aware weights and (ii) horizon weights:

$$\mathcal{L} = \sum_{t=1}^{T}\sum_{h=1}^{H} \alpha_{t+h}\, \omega_h \cdot \ell_\delta(\hat{y}_{t+h} - y_{t+h}), \tag{S16}$$

where $\omega_h \geq 0$ controls the relative importance of different forecast horizons (e.g., $\sum_{h=1}^{H} \omega_h = 1$), and $\alpha_{t+h} \in [0,1]$ down-weights targets that are likely to be revised due to reporting delay/backfill (with $\alpha_{t+h} = 0$ indicating an unavailable label). The Huber loss is defined as

$$\ell_\delta(e) = \begin{cases} \frac{1}{2}e^2, & |e| \leq \delta, \\ \delta\left(|e| - \frac{1}{2}\delta\right), & \text{otherwise,} \end{cases} \tag{S17}$$

with $e = \hat{y}_{t+h} - y_{t+h}$ and threshold $\delta > 0$.

During optimization, the LLM backbone and its input embedding matrix remain fully frozen. We update only the Temporal State Encoder, the State Prompt Adapter, the Protocol Anchor Bank (including the anchor summarizer and attention projections), and the prediction head, yielding a parameter-efficient adaptation for epidemic forecasting under heterogeneous surveillance systems. The frozen design should not be interpreted as implying that the LLM is incidental. Rather, the LLM remains central as the semantic integration backbone of the framework. We keep it frozen for efficiency and parameter-efficient adaptation: task-specific learning is concentrated in the TSE, SPA, PAB, and prediction head, allowing the model to exploit the representational structure of a large pretrained backbone without the computational cost and instability of full fine-tuning.

5 APPENDIX C: EXPERIMENTAL DETAILS AND ADDITIONAL RESULTS

5.1 Datasets

We evaluate **EpiMap-LLM** on two widely used public-health surveillance benchmarks that represent complementary reporting regimes:

JHU CSSE COVID-19 (Daily). The Johns Hopkins CSSE repository provides daily reported epidemic counts at multiple geographic resolutions, which makes it suitable for studying cross-region heterogeneity and protocol variability (e.g., reporting delays and batch corrections). We follow the common practice in public-health forecasting and model a daily forecasting task using aligned regional time series Dong et al. (2022).

CDC Influenza Hospitalization Surveillance (CDC-IHA / FluSurv-NET, Weekly). This dataset reports weekly influenza-related hospitalization signals (and related surveillance indicators) across U.S. jurisdictions. A key challenge is that weekly surveillance is often subject to revision/backfill: early releases can be incomplete and are subsequently updated. This setting is therefore well-suited for evaluating

protocol-aware robustness Reich et al. (2019); McGowan et al. (2019). From a health information perspective, these surveillance streams are versioned: preliminary releases are revised over time, and model training/evaluation should reflect the data version available at the forecast time.

Preprocessing and splits. For each dataset, we standardize the target variable(s) and apply a rolling-origin evaluation protocol: at each cutoff time t, models observe the history up to t and predict future horizons. We use a validation window for early stopping and hyperparameter selection, and report results on a held-out test window at the end of the timeline. For CDC-IHA, we additionally construct a backfill-aware mask that excludes targets that are not yet finalized at the forecast time, to avoid leaking post-hoc revisions into training or evaluation. Where applicable, we align supervision to the data vintage available at each forecast origin to ensure a realistic, deployment-consistent evaluation under revisions.

5.2 Evaluation Metrics

We report point-forecast accuracy using two standard metrics in epidemic time-series evaluation: Mean Absolute Error (MAE) and Root Mean Squared Error (RMSE). Given a set of valid forecast targets $\{y_i\}_{i=1}^N$ and predictions $\{\hat{y}_i\}_{i=1}^N$, we compute:

$$\mathrm{MAE} = \frac{1}{N}\sum_{i=1}^{N}|y_i - \hat{y}_i|, \tag{S18}$$

$$\mathrm{RMSE} = \sqrt{\frac{1}{N}\sum_{i=1}^{N}(y_i - \hat{y}_i)^2}. \tag{S19}$$

5.3 Baselines

We compare EpiMap-LLM against forecasting baselines that span distinct modeling paradigms and inductive biases. The goal is not to exhaust all variants, but to cover representative "families" that are commonly used for real-world time-series forecasting: classical statistical baselines, long-horizon Transformers, continuous-time neural dynamics for irregular sampling, graph-based patch modeling, and a large-scale foundation forecaster.

Classical statistical baselines. To ground comparisons in public-health practice, we include a simple seasonal/persistence baseline (e.g., last-week value for weekly series, or a 7-day seasonal naive for daily series) and a classical statistical forecaster (e.g., ARIMA or ETS) as low-complexity references. These baselines reflect widely used operational heuristics and provide a transparent lower bound for deployment settings.

Long-horizon Transformer forecasters. We include **Informer** Zhou et al. (2021) and **Autoformer** Wu et al. (2021) as canonical Transformer variants designed for long-range forecasting. Informer improves efficiency for long contexts via ProbSparse self-attention (reducing the quadratic attention cost) and a distilling operation that progressively compresses the encoder representations, paired with a decoder that generates the prediction horizon in a single forward pass rather than step-by-step autoregression Zhou et al. (2021). Autoformer departs from point-wise attention by integrating series decomposition as a core architectural block (progressively separating trend/seasonal components across layers) and replacing standard self-attention with an Auto-Correlation mechanism that aggregates dependencies at the sub-series level to better capture periodic structure Wu et al. (2021).

Continuous-time / neural dynamics models. To account for reporting gaps and irregular sampling patterns that are common in surveillance data, we include three continuous-time baselines. **ODE-RNN** Rubanova et al. (2019) combines a discrete RNN update with an ODE solver between observation times, yielding a latent trajectory that evolves continuously across irregular intervals. **Neural-CDE** Kidger et al. (2020) models the observed path as a control signal and learns hidden dynamics through controlled differential equations, which is particularly suitable when observations are asynchronous or unevenly spaced. **ContiFormer** Chen et al. (2023) integrates continuous-time dynamics with Transformer-style attention, using an ODE-based transition kernel between observations together with attention blocks for relational modeling across time.

Graph-based patch model. We include **T-PatchGNN** Zhang et al. (2024), which converts a time series into a graph of overlapping temporal patches and applies message passing to capture both local continuity and longer-range dependencies. This patch-graph construction provides a complementary bias to sequence attention, and has been shown to be effective in sparse or low-signal regimes.

Foundation forecaster. We include **TimesFM** Das et al. (2024), a decoder-only time-series foundation model pretrained on a very large corpus of real-world time points. TimesFM serves as a strong reference for general-purpose forecasting capacity, especially in transfer settings where pretrained representations can provide robust priors.

Why these baselines. Together, the above baselines provide a compact but high-coverage comparison set: (i) classical statistical baselines (seasonal naive; ARIMA/ETS), (ii) long-horizon Transformers (Informer/Autoformer), (iii) continuous-time neural dynamics (ContiFormer/Neural-CDE/ODE-RNN), (iv) graph-based temporal structure (T-PatchGNN), and (v) large-scale foundation forecasting (TimesFM). This diversity allows us to test whether EpiMap-LLM's protocol-aware semantic conditioning offers benefits beyond any single architectural prior.

5.4 Implementation Details

Model configuration. Unless stated otherwise, the LLM backbone and its input embedding matrix are kept fully frozen. We train only the lightweight components in Fig. S1: (i) the Temporal State Encoder, (ii) the State Prompt Adapter, (iii) the Protocol Anchor Bank (including anchor summarization and attention projections), and (iv) the prediction head.

Training and optimization. All models are optimized with AdamW and early stopping based on validation MAE. For EpiMap-LLM, the training objective uses a Huber loss with horizon weights $\{\omega_h\}$ and backfill-aware weights $\{\alpha_{t+h}\}$ (Section 4.7). We set $\sum_{h=1}^{H} \omega_h = 1$ and use uniform horizon weighting by default unless otherwise specified. The backfill weights down-weight targets that are likely to be revised (and mask unavailable labels). We tune the learning rate in $\{1\text{e}{-3},\ 3\text{e}{-4},\ 1\text{e}{-4}\}$ and dropout in $\{0.1,\ 0.3,\ 0.5\}$, selecting hyperparameters on the validation window. We apply gradient clipping to stabilize training and use a cosine learning-rate schedule when optimization is sensitive under distribution shifts.

Forecasting horizons and evaluation protocol. We evaluate multiple forecasting horizons from short- to medium-range settings using a rolling-origin protocol with a fixed context length. For JHU (daily), we report results for $H = \{1, 3, 7, 14\}$ days ahead; for CDC-IHA (weekly), we report results for $H = \{1, 2, 3, 4\}$ weeks ahead. All baselines follow the same train/validation/test splits, forecasting horizons, and preprocessing pipeline to ensure a fair comparison. For datasets with revisions/backfill, evaluation is conducted only on valid targets that are consistent with the backfill-aware labeling protocol.

Hardware and reproducibility. Experiments are implemented in PyTorch and run on a single RTX 409 NVIDIA GPU. We report the mean and standard deviation over multiple random seeds for all trainable models and release configuration details to support reproducibility. All experiments use publicly available, aggregated surveillance data and do not involve individual-level records.

REFERENCES

Ansari, A. F., Stella, L., Türkmen, A. C., Zhang, X., Mercado, P., Shen, H., et al. (2024). Chronos: Learning the language of time series. CoRR abs/2403.07815. doi:10.48550/ARXIV.2403.07815

Chang, C., Peng, W.-C., and Chen, T.-F. (2024). Llm4ts: Two-stage fine-tuning for time-series forecasting with pre-trained llms. In Proceedings of the AAAI Conference on Artificial Intelligence. vol. 38, 11314–11322

Chen, Y., Ren, K., Wang, Y., Fang, Y., Sun, W., and Li, D. (2023). Contiformer: Continuous-time transformer for irregular time series modeling. In Advances in Neural Information Processing Systems (NeurIPS)

Cramer, E. Y., Huang, Y., Wang, Y., Ray, E. L., Cornell, M., Bracher, J., et al. (2022). The united states covid-19 forecast hub dataset. Scientific Data 9, 462. doi:10.1038/s41597-022-01517-w

Das, A., Kong, W., Sen, R., and Zhou, Y. (2024). A decoder-only foundation model for time-series forecasting. In Proceedings of the 41st International Conference on Machine Learning, eds. R. Salakhutdinov, Z. Kolter, K. Heller, A. Weller, N. Oliver, J. Scarlett, and F. Berkenkamp (PMLR), vol. 235 of Proceedings of Machine Learning Research, 10148–10167

Dong, E., Ratcliff, J., Goyea, T. D., Katz, A., Lau, R., Ng, T. K., et al. (2022). The johns hopkins university center for systems science and engineering covid-19 dashboard: data collection process, challenges faced, and lessons learned. The Lancet Infectious Diseases 22, e370–e376. doi:10.1016/S1473-3099(22)00434-0

Gao, S., Shi, T. K., Ning, W., et al. (2024). Units: Building a unified time series model. In International Conference on Learning Representations

Höhle, M. and an der Heiden, M. (2014). Bayesian nowcasting during the stec o104:h4 outbreak in germany, 2011. Biometrics 70, 993–1002. doi:10.1111/biom.12194

Jin, M., Wang, S., Ma, L., Chu, Z., Zhang, J., Shi, X., et al. (2023). Time-llm: Time series forecasting by reprogramming large language models. arXiv preprint arXiv:2310.01728

Kidger, P., Morrill, J., Foster, J., and Lyons, T. J. (2020). Neural controlled differential equations for irregular time series. In Advances in Neural Information Processing Systems 33 (NeurIPS 2020)

McGowan, C. J., Biggerstaff, M., Johansson, M., Apfeldorf, K. M., Ben-Nun, M., Brooks, L., et al. (2019). Collaborative efforts to forecast seasonal influenza in the united states, 2015–2016. Scientific Reports 9, 683. doi:10.1038/s41598-018-36361-9

Nie, Y., Nguyen, N. H., Sinthong, P., and Kalagnanam, J. (2023). A time series is worth 64 words: Long-term forecasting with transformers. In The Eleventh International Conference on Learning Representations (ICLR 2023) (OpenReview.net)

Ray, E. L., Brooks, L. C., Bien, J., Biggerstaff, M., Bosse, N. I., Bracher, J., et al. (2023). Comparing trained and untrained probabilistic ensemble forecasts of covid-19 cases and deaths in the united states. International Journal of Forecasting 39, 1366–1383. doi:10.1016/j.ijforecast.2022.06.005

Reich, N. G., Brooks, L. C., Fox, S. J., Kandula, S., McGowan, C. J., Moore, E., et al. (2019). A collaborative multiyear, multimodel assessment of seasonal influenza forecasting in the united states. Proceedings of the National Academy of Sciences 116, 3146–3154. doi:10.1073/pnas.1812594116

Rubanova, Y., Chen, T. Q., and Duvenaud, D. (2019). Latent ordinary differential equations for irregularly-sampled time series. In Advances in Neural Information Processing Systems 32 (NeurIPS 2019). 5321–5331

Wu, H., Xu, J., Wang, J., and Long, M. (2021). Autoformer: Decomposition transformers with auto-correlation for long-term series forecasting. In Advances in Neural Information Processing Systems. vol. 34, 22419–22430

Xue, H. and Salim, F. D. (2023). Promptcast: A new prompt-based learning paradigm for time series forecasting. IEEE Transactions on Knowledge and Data Engineering

Zeroual, A., Harrou, F., Dairi, A., and Sun, Y. (2020). Deep learning methods for forecasting covid-19 time-series data: A comparative study. Chaos, solitons & fractals 140, 110121

Zhang, W., Yin, C., Liu, H., Zhou, X., and Xiong, H. (2024). Irregular multivariate time series forecasting: A transformable patching graph neural networks approach. In Proceedings of the 41st International Conference on Machine Learning (PMLR), 63574–63595

Zhou, H., Zhang, S., Peng, J., Zhang, S., Li, J., Xiong, H., et al. (2021). Informer: Beyond efficient transformer for long sequence time-series forecasting. In Thirty-Fifth AAAI Conference on Artificial Intelligence (AAAI 2021). 11106–11115. doi:10.1609/AAAI.V35I12.17325

Zivkovic, M., Bacanin, N., Venkatachalam, K., Nayyar, A., Djordjevic, A., Strumberger, I., et al. (2021). Covid-19 cases prediction by using hybrid machine learning and beetle antennae search approach. Sustainable cities and society 66, 102669
